# Supplementary material for: Increased immunogen valency improves the maturation of vaccine-elicited HIV-1 VRC01-class antibodies
Source: bioRxiv. 2025 Mar 14:2025.03.13.642975. Preprint. [Version 1] doi: 10.1101/2025.03.13.642975 (PMC11952507; doi:10.1101/2025.03.13.642975)
Supplement: Supplement 1 [file NIHPP2025.03.13.642975v1-supplement-1.pdf]

bioRxiv preprint doi: https://doi.org/10.1101/2025.03.13.642975; this version posted March 14, 2025. The copyright holder for this preprint (which was not certified by peer review) is the author/funder, who has granted bioRxiv a license to display the preprint in perpetuity. It is made available under aCC-BY 4.0 International license.

Fig S1. **Heavy chain/Light chain sequence analysis at the indicated time points in both NP groups.** Pie charts indicate HC (A, B) and LC (C to F) characteristics from individually sorted Env-specific B cells from pooled mouse samples. The number of HC and LC sequences analyzed is shown in the middle of each pie chart. (A) VH-gene usage, (B) HCs with the H35N mutation are shown. (C) aa length of the CDRL3 domains in the LC, (D) LC-gene usage, where shades of grey/black slices represent non 5-aa long CDRL3s and blue indicates other 5-aa CDRL3s. (E) Presence of Glu_{96} within the LC sequences with 5-aa long CDRL3 domains, and (F) Logo plot showing CDRL3 region from the two NP groups at the indicated time points.

Fig S2. **Binding curves of VRC01-class mAbs generated at different timepoints in the two NP groups.** mAbs were evaluated against the indicated soluble monomeric Envs and their knock-outs (KO) using BLI assay. mVRC01 (solid pink line) and glVRC01 (solid cyan line) were included as internal controls. Black dotted lines indicate end of association and dissociation phases.

Fig S3. **Binding curves of VRC01-class mAbs generated in the two NP groups against heterologous WT.Core Envs**. mVRC01 (solid pink line) and glVRC01 (solid cyan line) were included as internal controls. Black dotted lines indicate end of association and dissociation phases.

Fig S4. **Binding curves of VRC01-class mAbs generated in the two NP groups against indicated variants of 426c SOSIP**. mVRC01 (solid pink line) and glVRC01 (solid cyan line) were included as internal controls. Black dotted lines indicate end of association and dissociation phases.

bioRxiv preprint doi: https://doi.org/10.1101/2025.03.13.642975; this version posted March 14, 2025. The copyright holder for this preprint (which was not certified by peer review) is the author/funder, who has granted bioRxiv a license to display the preprint in perpetuity. It is made available under aCC-BY 4.0 International license.

Fig S5. **Number of amino acid changes in the HC and LC of paired sequences at week 2, week 23, and week 25, from both NPs groups**. Each circle represents a paired sequence and '*' indicates significant differences using Kruskal-Wallis test.

Fig S6. (A) Phylogenetic tree including both paired and unpaired observed sequences in the Fer NP group. Timepoints are colored as indicated (with inferred ancestral sequences in grey), and antibodies chosen for synthesis are labeled in red. (B) Identification of single-timepoint subtrees and calculation of the resulting subtree size (top) and ancestor distance (bottom).

Fig S7. **Comparison of sequence alignment of post-boost VRC01-class mAbs from both NP groups**. Germline VH1-2*02 and κ8-30*01 sequences are used as reference for alignment, and CDRs are highlighted in red. Red ovals highlight the residues commonly present in mature VRC01-class antibodies.

Table S1. **Information on the VRC01-class mAbs isolated at the indicated time points in the two NP groups.** A total of 33 VRC01-class mAbs were successfully generated from the immunized animals.

Table S2. **HC/LC sequences including that of the VRC01-class antibodies isolated after the final immunization, related to Fig. 4.** Amino acid sequences are aligned to the V genes from which they are derived, and CDRs are highlighted in red.

Figure S1, Related to Fig 3

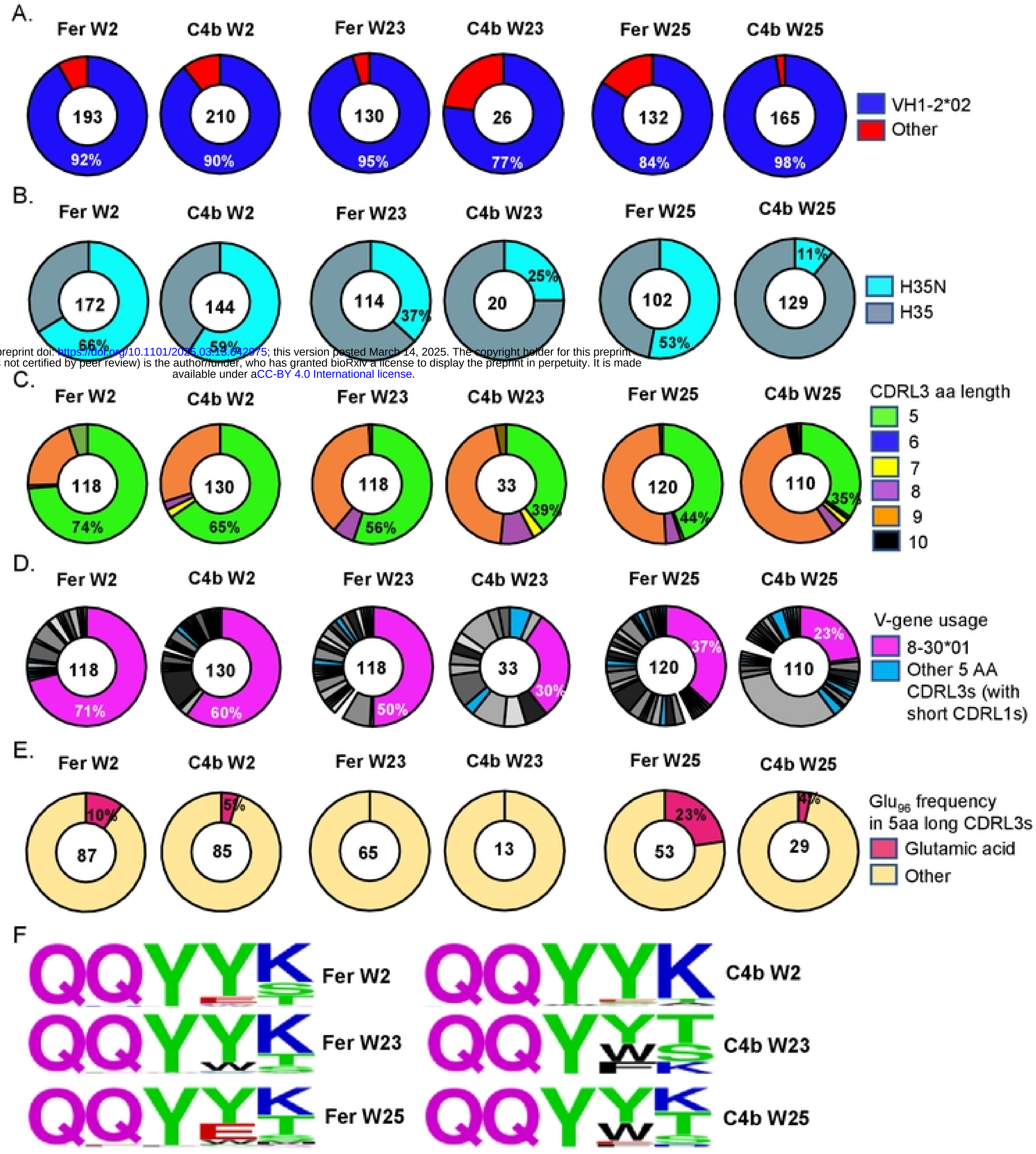

bioRxiv preprint doi: https://doi.org/10.1101/2025.03.13.642975; this version posted March 14, 2025. The copyright holder for this preprint (which was not certified by peer review) is the author/funder, who has granted bioRxiv a license to display the preprint in perpetuity. It is made available under aCC-BY 4.0 International license.

Figure S2, Related to Figure 4

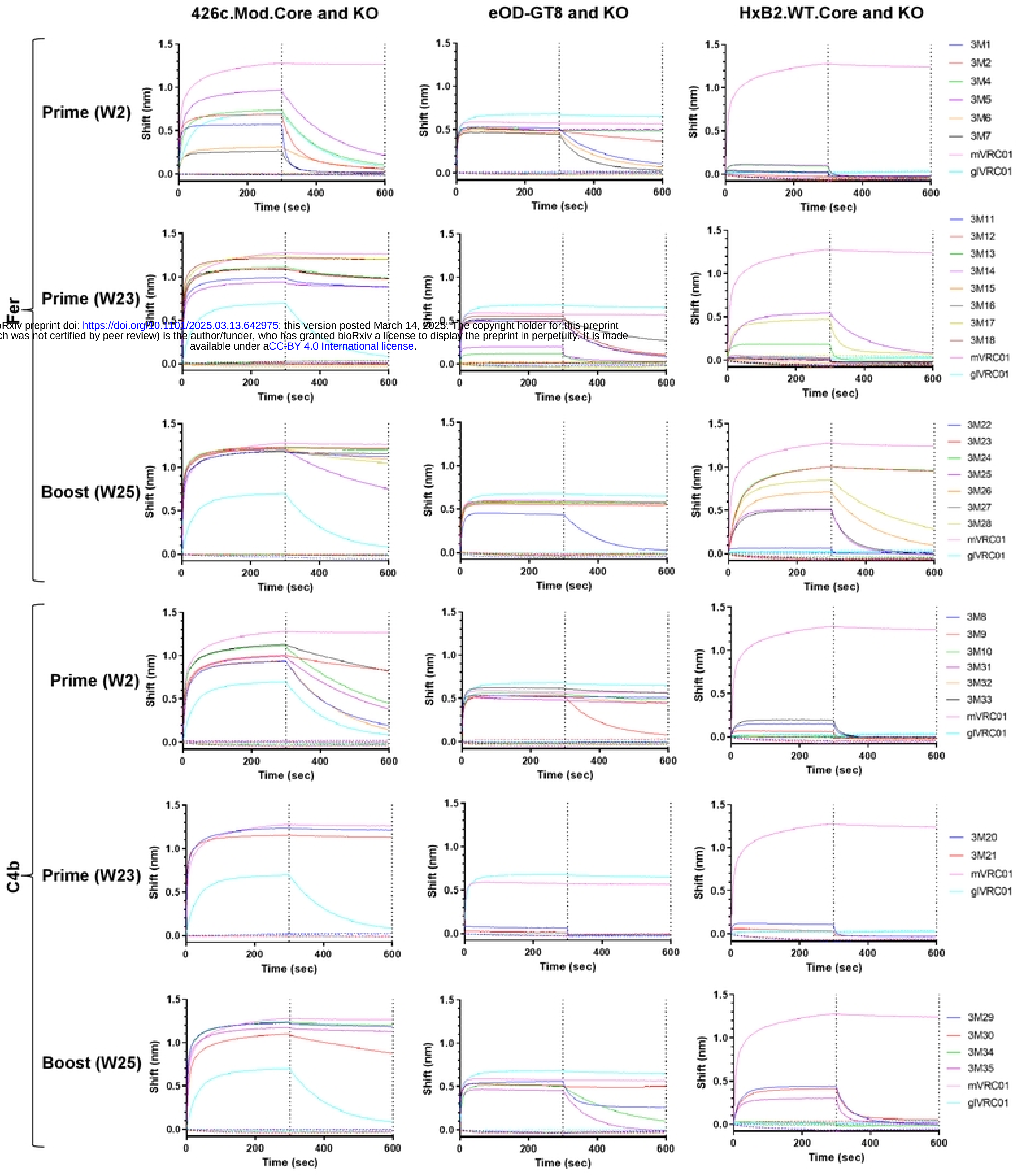

bioRxiv preprint doi: https://doi.org/10.1101/2025.03.13.642975; this version posted March 14, 2025. The copyright holder for this preprint (which was not certified by peer review) is the author/funder, who has granted bioRxiv a license to display the preprint in perpetuity. It is made available under aCC-BY 4.0 International license.

Figure S3, Related to Figure 5

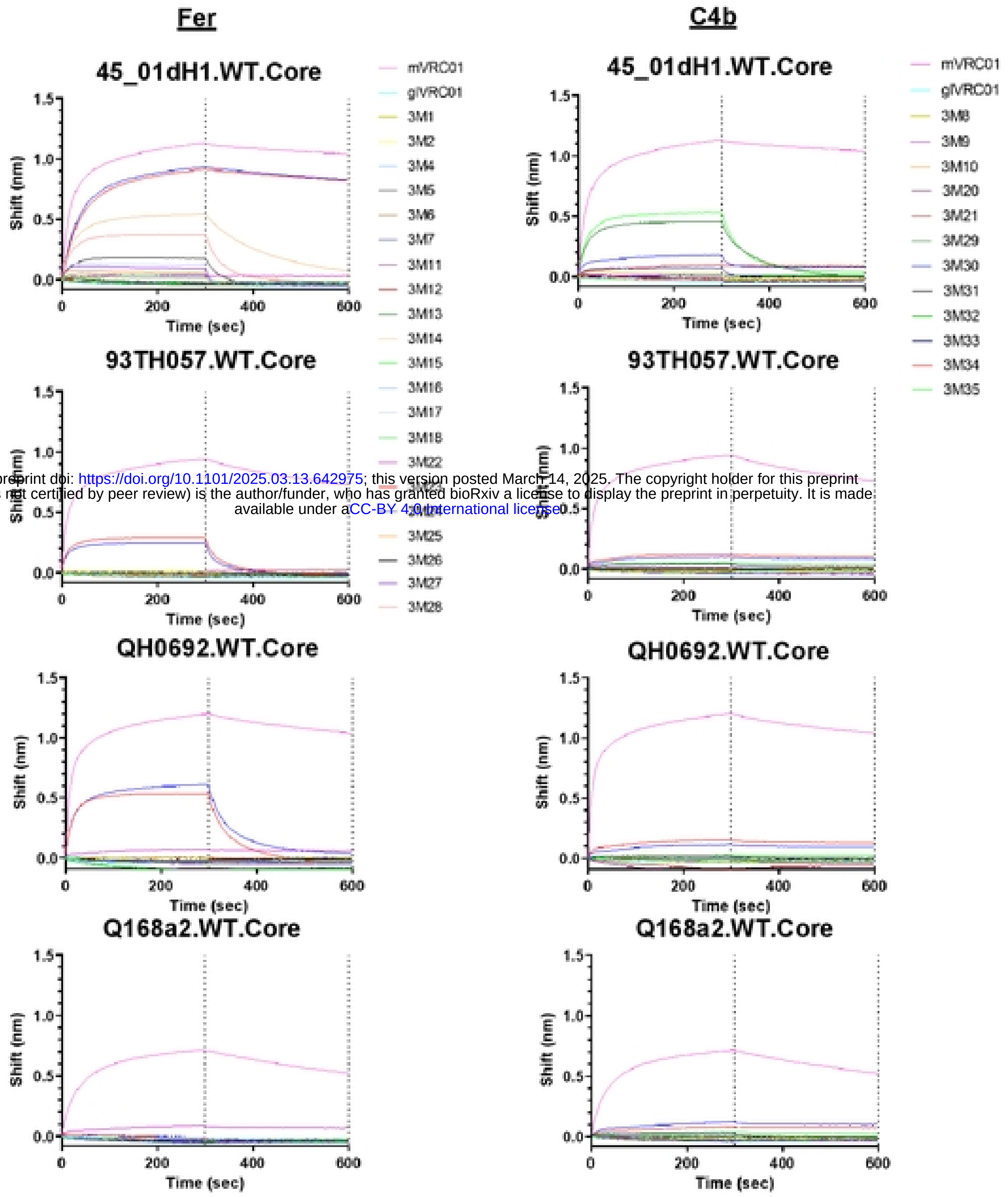

bioRxiv preprint doi: https://doi.org/10.1101/2025.03.13.642975; this version posted March 14, 2025. The copyright holder for this preprint (which was not certified by peer review) is the author/funder, who has granted bioRxiv a license to display the preprint in perpetuity. It is made available under aCC-BY 4.0 International license.

Figure S4, Related to Figure 5

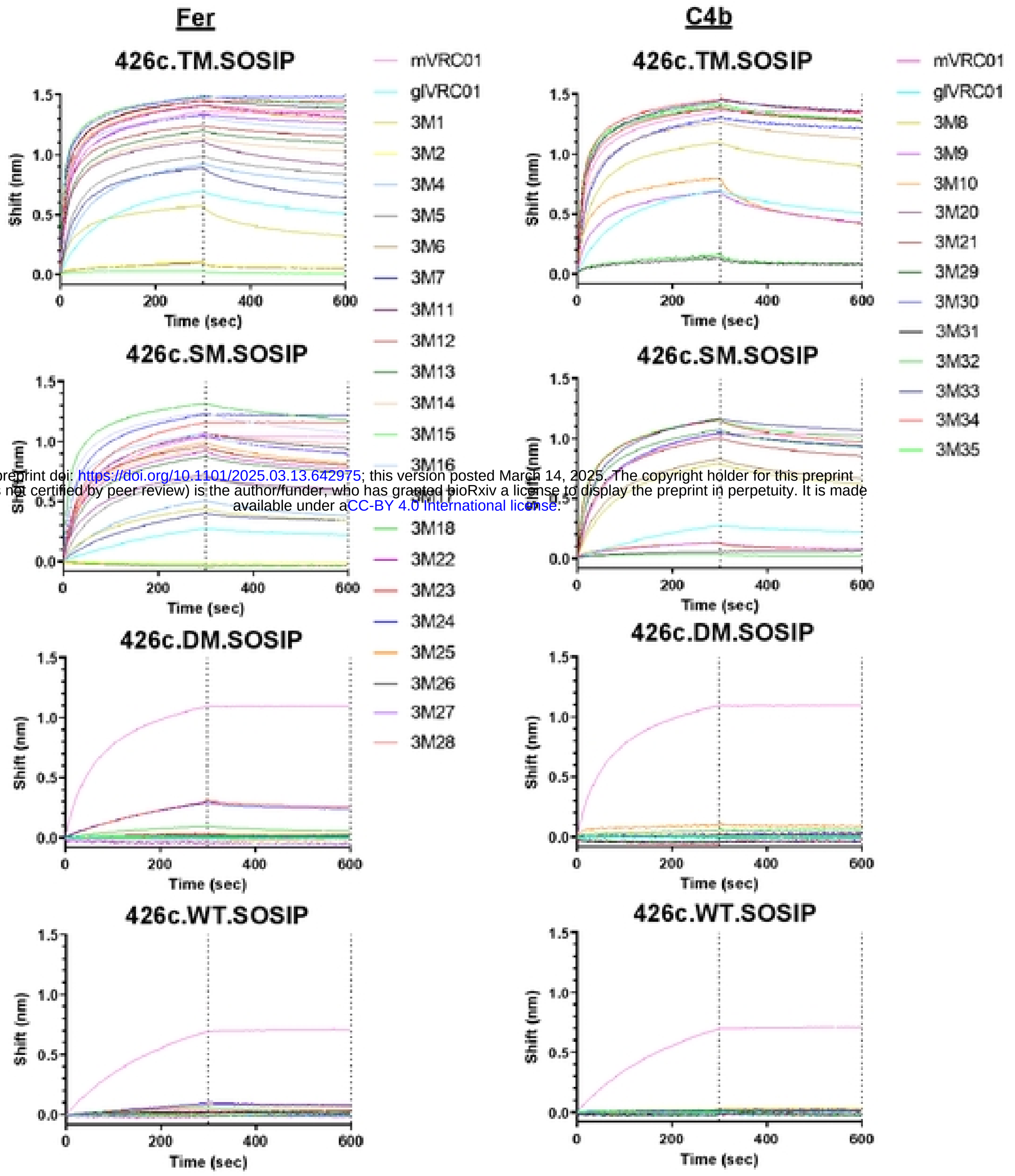

bioRxiv preprint doi: https://doi.org/10.1101/2025.03.13.642975; this version posted March 14, 2025. The copyright holder for this preprint (which was not certified by peer review) is the author/funder, who has granted bioRxiv a license to display the preprint in perpetuity. It is made available under aCC-BY 4.0 International license.

Figure S5, Related to Figure 6

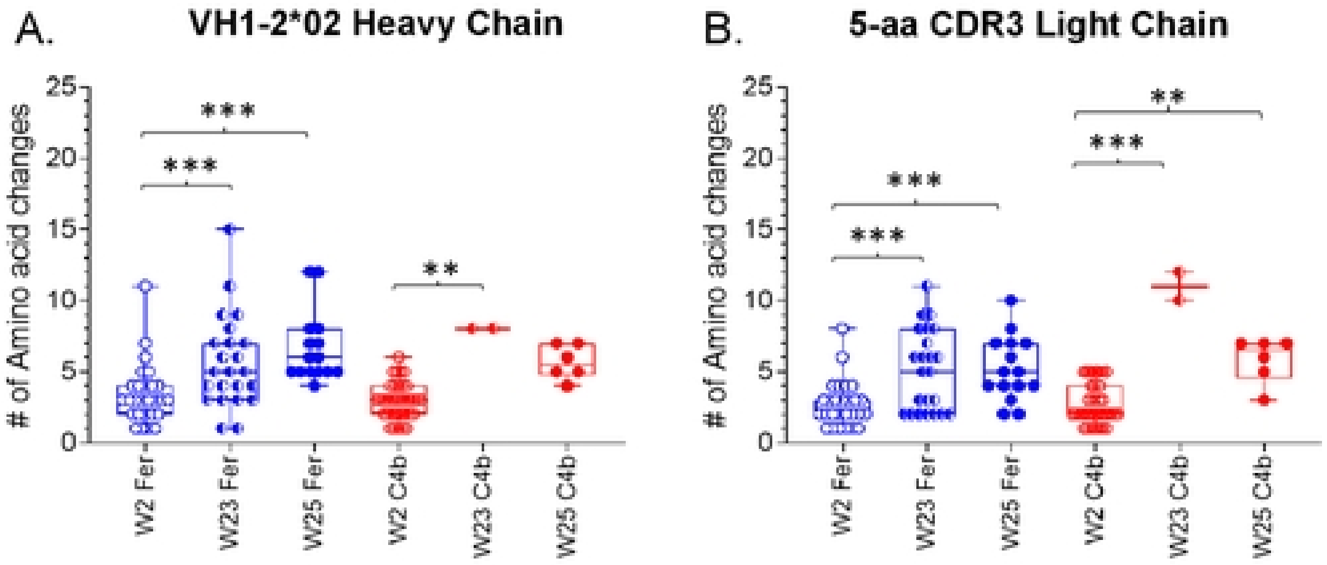

bioRxiv preprint doi: https://doi.org/10.1101/2025.03.13.642975; this version posted March 14, 2025. The copyright holder for this preprint (which was not certified by peer review) is the author/funder, who has granted bioRxiv a license to display the preprint in perpetuity. It is made available under aCC-BY 4.0 International license.

Figure S6, Related to Fig 7, 8

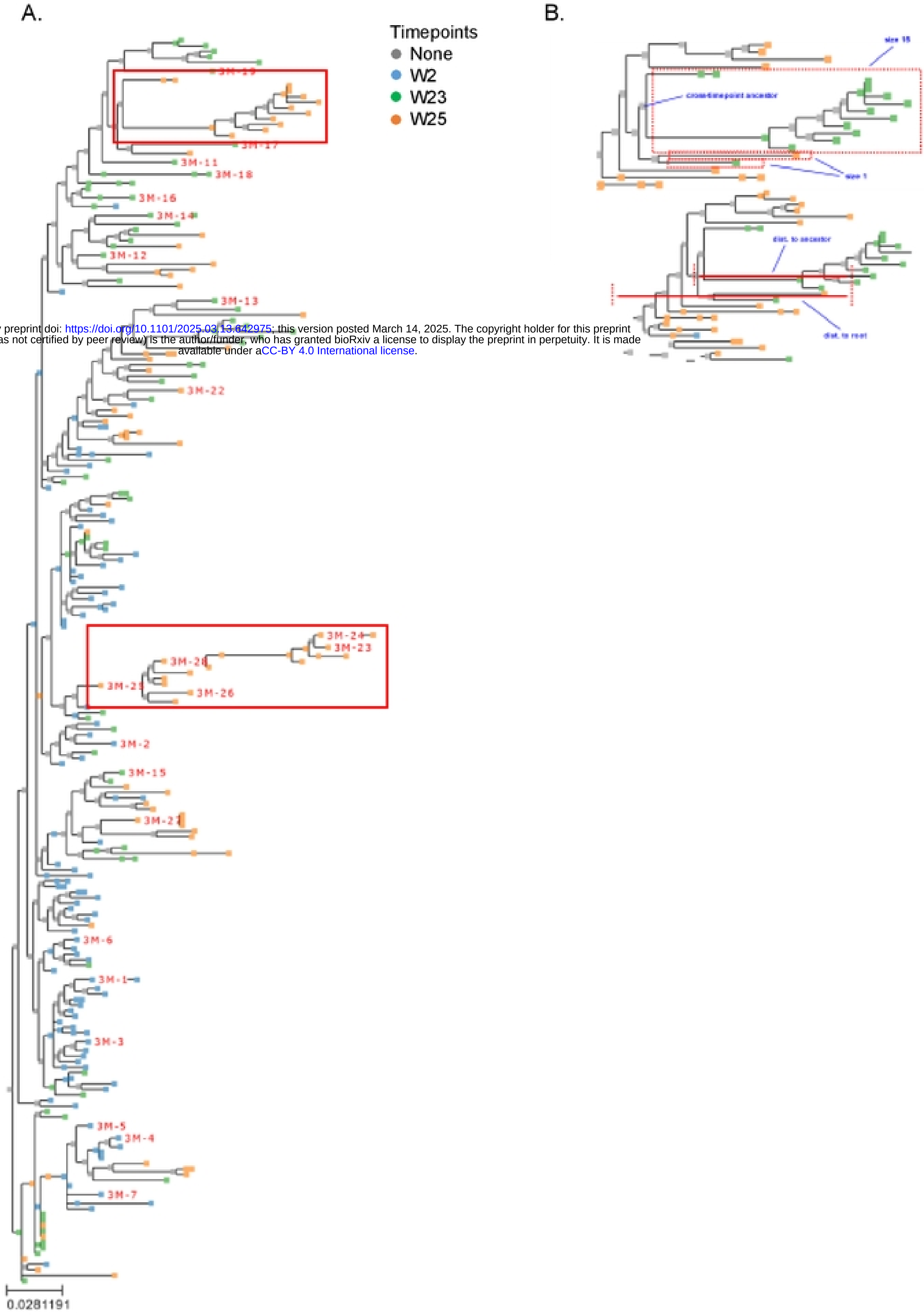

bioRxiv preprint doi: https://doi.org/10.1101/2025.03.13.642975; this version posted March 14, 2025. The copyright holder for this preprint (which was not certified by peer review) is the author/funder, who has granted bioRxiv a license to display the preprint in perpetuity. It is made available under aCC-BY 4.0 International license.

Figure S7, related to Figure 8

VH

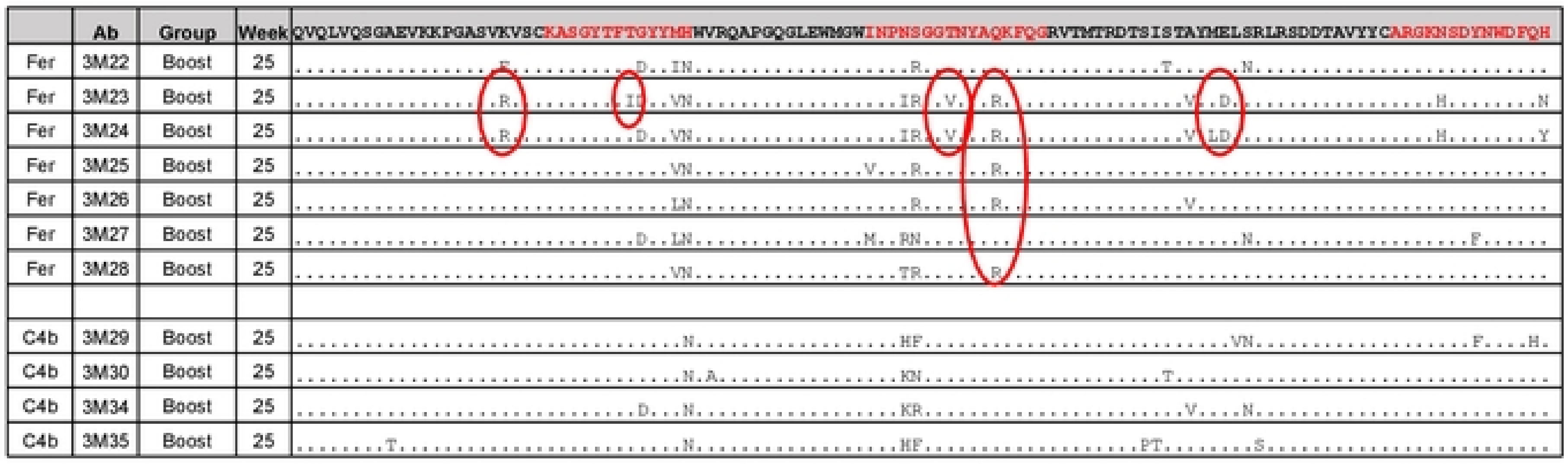

VL

bioRxiv preprint doi: https://doi.org/10.1101/2025.03.13.642975; this version posted March 14, 2025. The copyright holder for this preprint (which was not certified by peer review) is the author/funder, who has granted bioRxiv a license to display the preprint in perpetuity. It is made available under aCC-BY 4.0 International license.

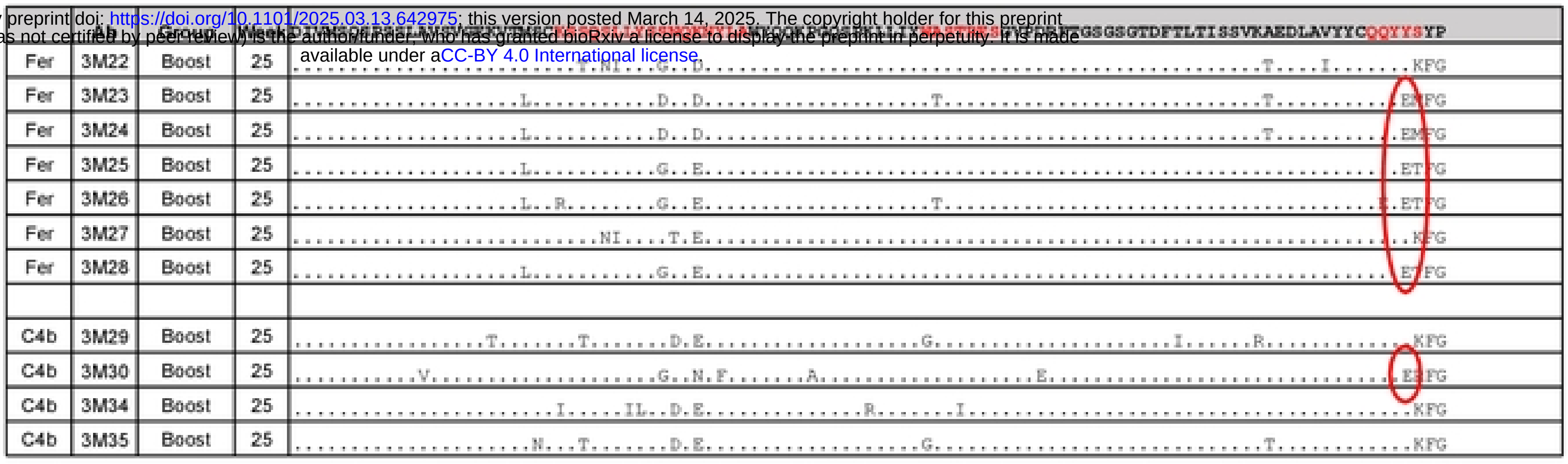

Table S1, related to Figure 4, 5, 6, and Table 1

Ab name	Group	Week	HC-V-GENE and allele	HC-J-GENE and allele	HC-AA JUNCTION	LC-V-GENE and allele	LC-J-GENE and allele	LC-AA JUNCTION
**3M1**	Prime-Fer	2	Homsap IGHV1-2*02	Homsap IGHJ1*01	CARGKTSDYNWDFQHW	Musmus IGKV8-30*01	Musmus IGKJ2*03	CQQYYNF
**3M2**	Prime-Fer	2	Homsap IGHV1-2*02	Homsap IGHJ1*01	CARGKNSDYNWDFQHW	Musmus IGKV8-30*01	Musmus IGKJ1*01	CQHYYKF
**3M3**	Prime-Fer	2	Homsap IGHV1-2*02	Homsap IGHJ1*01	CARGKHGDYNWDFQHW	Musmus IGKV8-30*01	Musmus IGKJ2*01	CQQYYKF
**3M4**	Prime-Fer	2	Homsap IGHV1-2*02	Homsap IGHJ1*01	CARGKNSNYNWDFQHW	Musmus IGKV8-30*01	Musmus IGKJ1*01	CQQYETF
**3M5**	Prime-Fer	2	Homsap IGHV1-2*02	Homsap IGHJ1*01	CARGKNSDYNWDFQHW	Musmus IGKV8-30*01	Musmus IGKJ1*01	CQQYETF
**3M6**	Prime-Fer	2	Homsap IGHV1-2*02	Homsap IGHJ1*01	CARGKNSDYNWDFQHW	Musmus IGKV8-30*01	Musmus IGKJ1*01	CQQYYKF
**3M7**	Prime-Fer	2	Homsap IGHV1-2*02	Homsap IGHJ1*01	CARGINSDYNWDFQHW	Musmus IGKV8-30*01	Musmus IGKJ1*01	CQQYETF
**3M8**	Prime-C4b	2	Homsap IGHV1-2*02	Homsap IGHJ1*01	CARGKNSDYNWDFQHW	Musmus IGKV8-30*01	Musmus IGKJ2*03	CQQYEAF
**3M9**	Prime-C4b	2	Homsap IGHV1-2*02	Homsap IGHJ1*01	CARGKNSDYNWDFQHW	Musmus IGKV8-30*01	Musmus IGKJ2*01	CQQYYKF
**3M10**	Prime-C4b	2	Homsap IGHV1-2*02	Homsap IGHJ2*01	CARGKNSEYHWEFXHW	Musmus IGKV8-30*01	Musmus IGKJ2*01	CQQYYKF
**3M31**	Prime-C4b	2	Homsap IGHV1-2*02	Homsap IGHJ1*01	CARGKNSDYNWDFQHW	Musmus IGKV8-30*01	Musmus IGKJ2*01	CQQYYKF
**3M32**	Prime-C4b	2	Homsap IGHV1-2*02	Homsap IGHJ1*01	CARGKNSDYNWDFQHW	Musmus IGKV8-30*01	Musmus IGKJ2*01	CQQYYKF
**3M33**	Prime-C4b	2	Homsap IGHV1-2*02	Homsap IGHJ1*01	CARGKNSDYNWDFQHW	Musmus IGKV8-30*01	Musmus IGKJ2*03	CQQYEAF
**3M11**	Prime-Fer	23	Homsap IGHV1-2*02	Homsap IGHJ1*01	CAKGKNSDYNWDFQHW	Musmus IGKV8-30*01	Musmus IGKJ2*01	CQLYYKF
**3M12**	Prime-Fer	23	Homsap IGHV1-2*02	Homsap IGHJ1*01	CARGKNSDYNWDFQHW	Musmus IGKV8-30*01	Musmus IGKJ1*01	CQQYYKF
**3M13**	Prime-Fer	23	Homsap IGHV1-2*02	Homsap IGHJ1*01	CARGKNSDYNWDFQHW	Musmus IGKV8-30*01	Musmus IGKJ4*01	CQQYYKF
**3M14**	Prime-Fer	23	Homsap IGHV1-2*02	Homsap IGHJ1*01	CARGKNSDYNWDFQHW	Musmus IGKV8-30*01	Musmus IGKJ1*01	CQQYWTF
**3M15**	Prime-Fer	23	Homsap IGHV1-2*02	Homsap IGHJ1*01	CARGKNSDYNWDFQHW	Musmus IGKV8-30*01	Musmus IGKJ1*01	CQQYYKF
**3M16**	Prime-Fer	23	Homsap IGHV1-2*02	Homsap IGHJ1*01	CARGKDSDYNWDFQHW	Musmus IGKV8-30*01	Musmus IGKJ5*01	CQQYYKF
**3M17**	Prime-Fer	23	Homsap IGHV1-2*02	Homsap IGHJ2*01	WAKRKNSDYNWDFXYW	Musmus IGKV8-30*01	Musmus IGKJ1*01	CQQYYKF
**3M18**	Prime-Fer	23	Homsap IGHV1-2*02	Homsap IGHJ1*01	CARGKDSDYNWDFQHW	Musmus IGKV8-30*01	Musmus IGKJ2*01	CQQYYKF
**3M19**	Prime-Fer	23	Homsap IGHV1-2*02	Homsap IGHJ1*01	CARGKDSDYNWDFQHW	Musmus IGKV8-30*01	Musmus IGKJ1*01	CQQYYKF
**3M20**	Prime-C4b	23	Homsap IGHV1-2*02	Homsap IGHJ1*01	CAKGKNSDYNWDFQHW	Musmus IGKV8-30*01	Musmus IGKJ2*03	CQQYYKF
**3M21**	Prime-C4b	[illegible]	[illegible]	[illegible]	[illegible]	[illegible]	Musmus IGKJ2*03	CQQYYKF
**[illegible]**	[illegible]	[illegible]	[illegible]	[illegible]	[illegible]	[illegible]	Musmus IGKJ4*01	CQQYYKF
**3M23**	Boost-Fer	25	Homsap IGHV1-2*02	Homsap IGHJ1*01	CARGKHSDYNWDFQNW	Musmus IGKV8-30*01	Musmus IGKJ1*01	CQQYEMF
**3M24**	Boost-Fer	25	Homsap IGHV1-2*02	Homsap IGHJ1*01	CARGKHSDYNWDFQYW	Musmus IGKV8-30*01	Musmus IGKJ1*01	CQQYEMF
**3M25**	Boost-Fer	25	Homsap IGHV1-2*02	Homsap IGHJ1*01	CARGKNSDYNWDFQHW	Musmus IGKV8-30*01	Musmus IGKJ1*01	CQQYETF
**3M26**	Boost-Fer	25	Homsap IGHV1-2*02	Homsap IGHJ1*01	CARGKNSDYNWDFQHW	Musmus IGKV8-30*01	Musmus IGKJ1*01	CQEYETF
**3M27**	Boost-Fer	25	Homsap IGHV1-2*02	Homsap IGHJ1*01	CARGKNSDFNWDFQHW	Musmus IGKV8-30*01	Musmus IGKJ1*01	CQQYYKF
**3M28**	Boost-Fer	25	Homsap IGHV1-2*02	Homsap IGHJ1*01	CARGKNSDYNWDFQHW	Musmus IGKV8-30*01	Musmus IGKJ1*01	CQQYETF
**3M29**	Boost-C4b	25	Homsap IGHV1-2*02	Homsap IGHJ1*01	CARGKNSDFNWDFHHW	Musmus IGKV8-30*01	Musmus IGKJ2*01	CQQYYKF
**3M30**	Boost-C4b	25	Homsap IGHV1-2*02	Homsap IGHJ1*01	CARGKNSDYNWDFQHW	Musmus IGKV8-30*01	Musmus IGKJ2*03	CQQYERF
**3M34**	Boost-C4b	25	Homsap IGHV1-2*02	Homsap IGHJ1*01	CARGKNSDYNWDFQHW	Musmus IGKV8-30*01	Musmus IGKJ1*01	CQQYYKF
**3M35**	Boost-C4b	25	Homsap IGHV1-2*02	Homsap IGHJ1*01	CARGKNSDYNWDFQHW	Musmus IGKV8-30*01	Musmus IGKJ5*01	CQQYYKF

bioRxiv preprint doi: https://doi.org/10.1101/2025.03.13.642975; this version posted March 14, 2025. The copyright holder for this preprint (which was not certified by peer review) is the author/funder, who has granted bioRxiv a license to display the preprint in perpetuity. It is made available under aCC-BY 4.0 International license.
